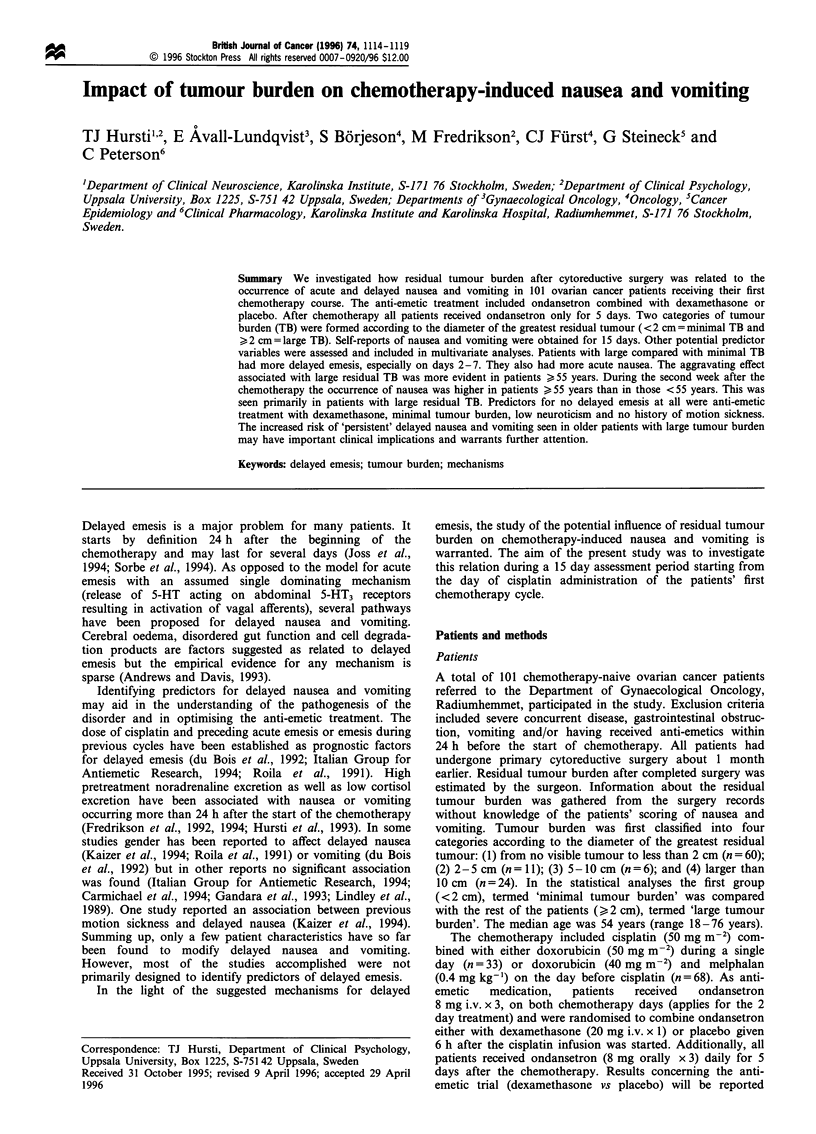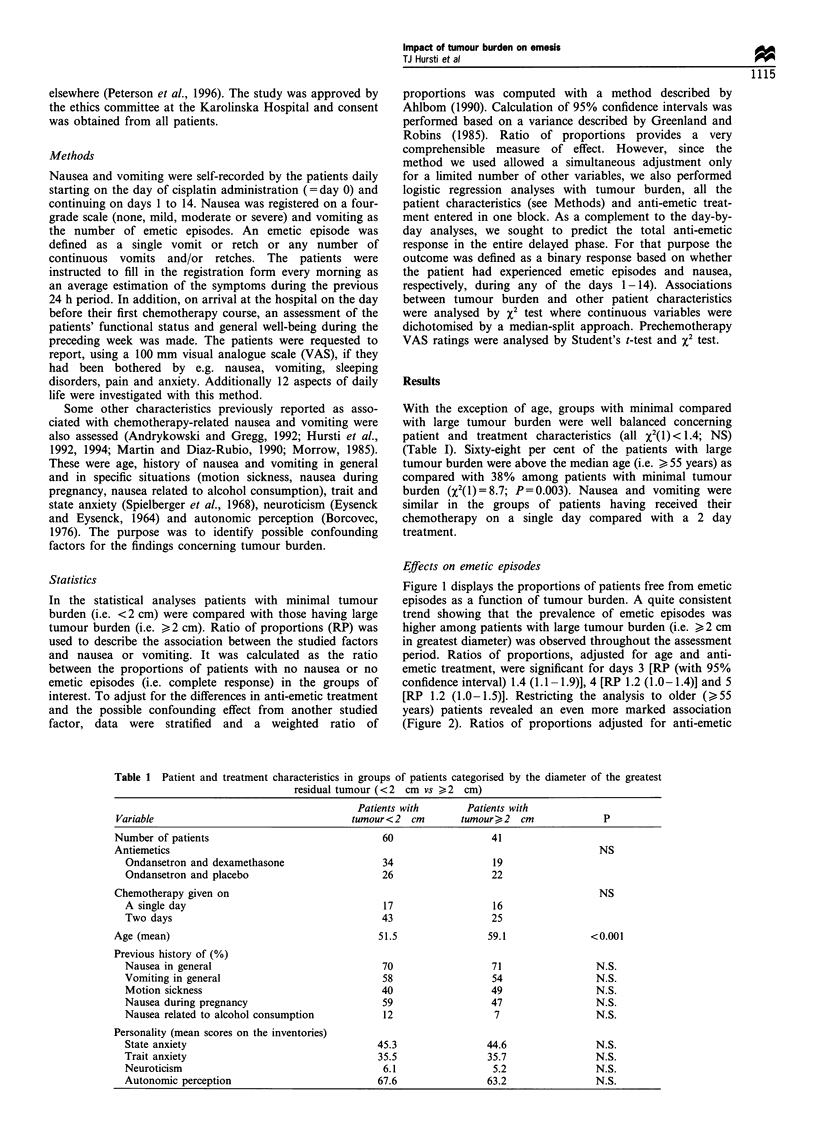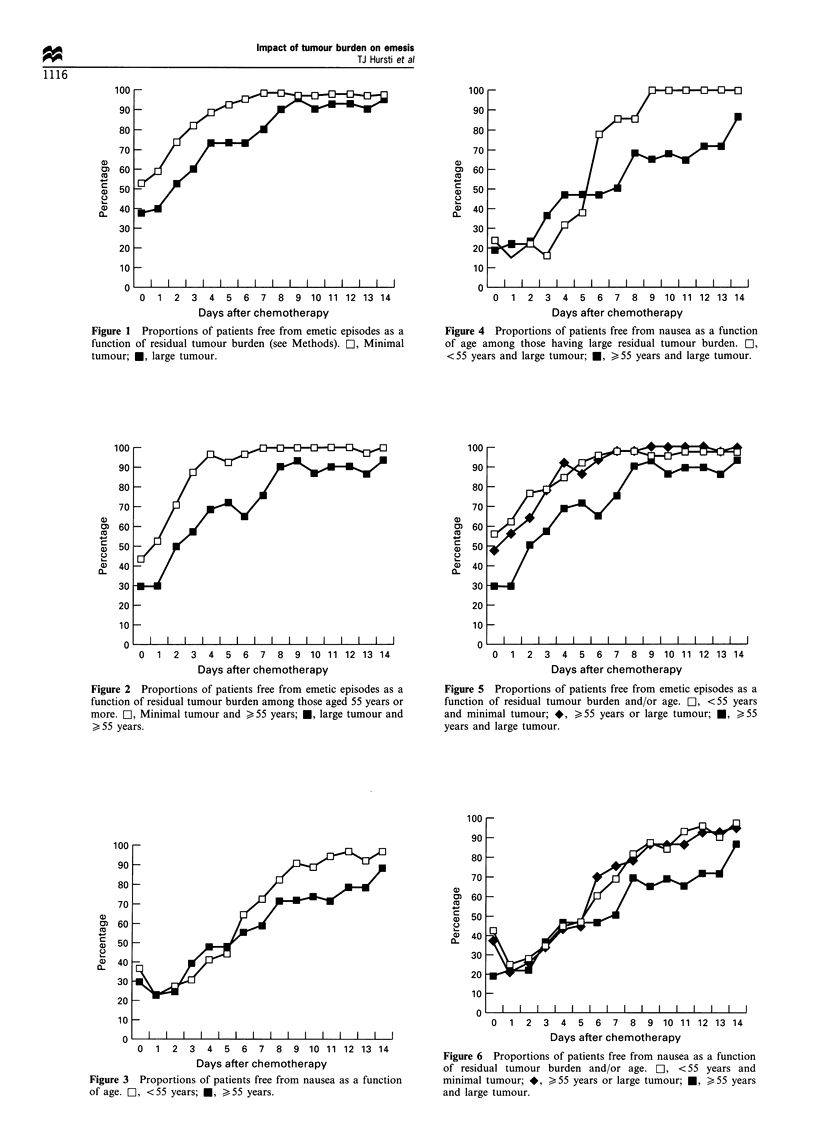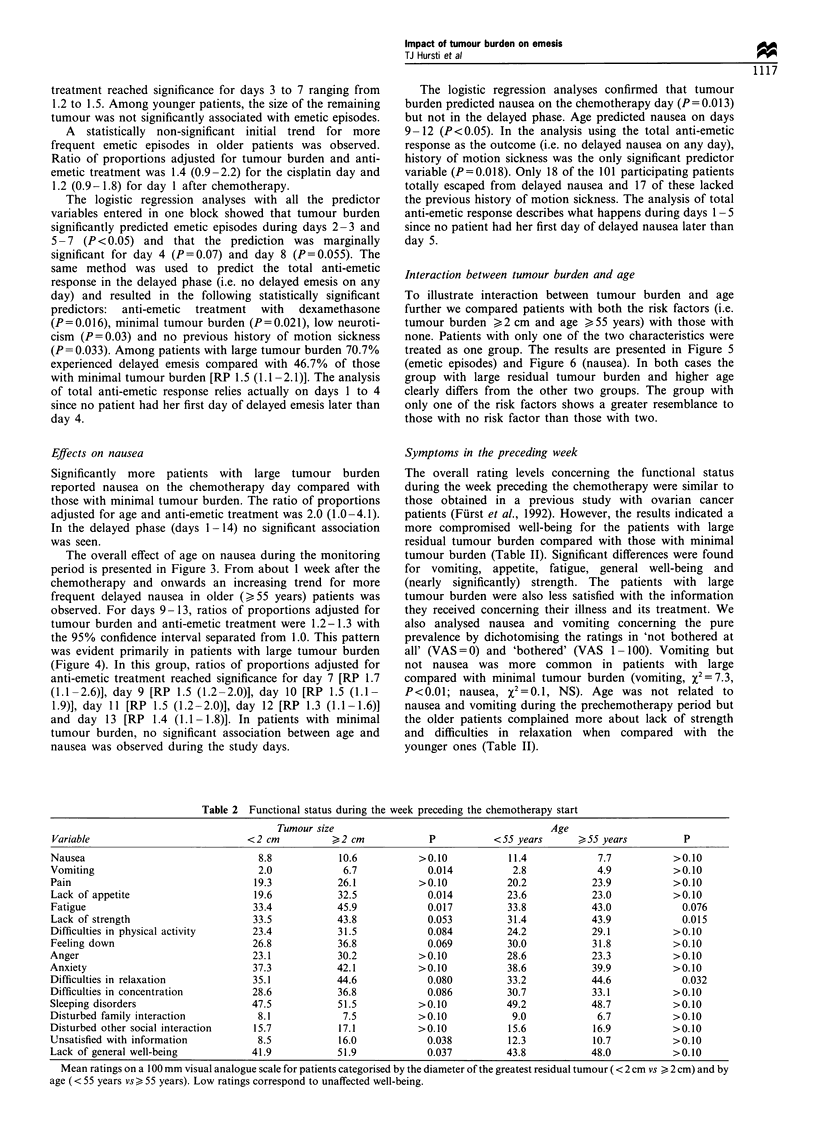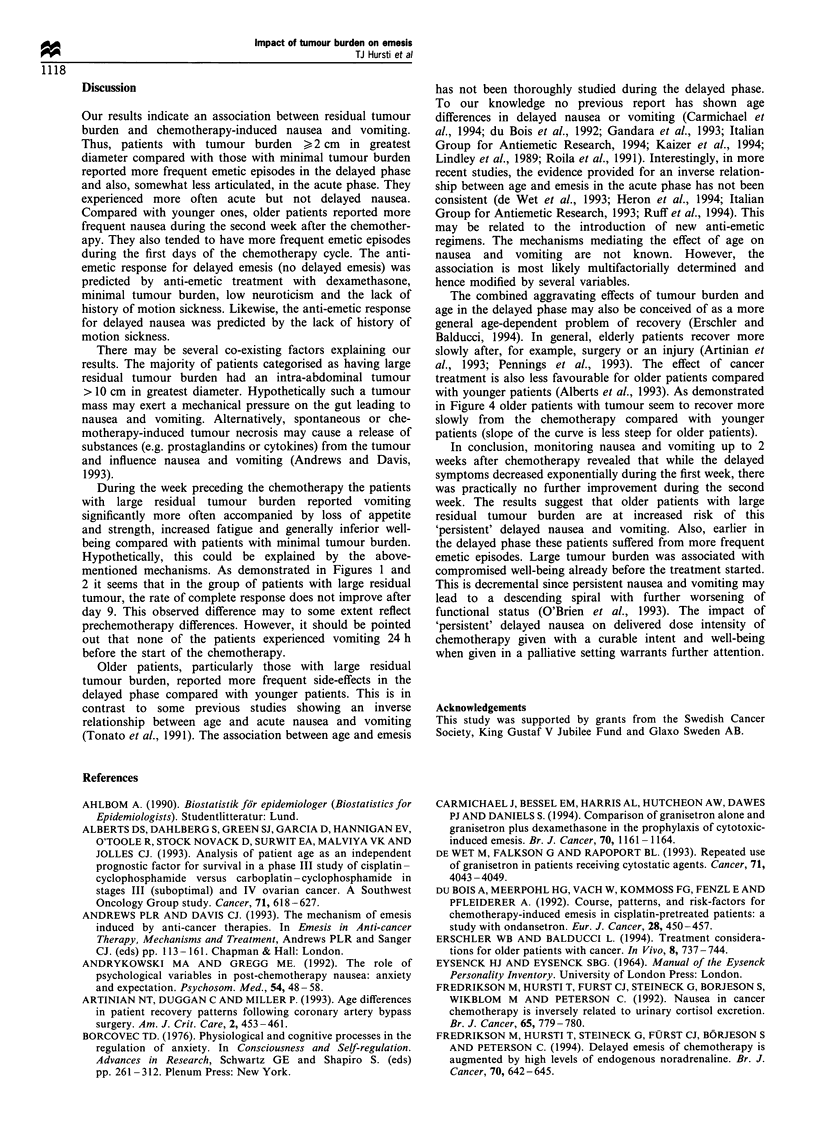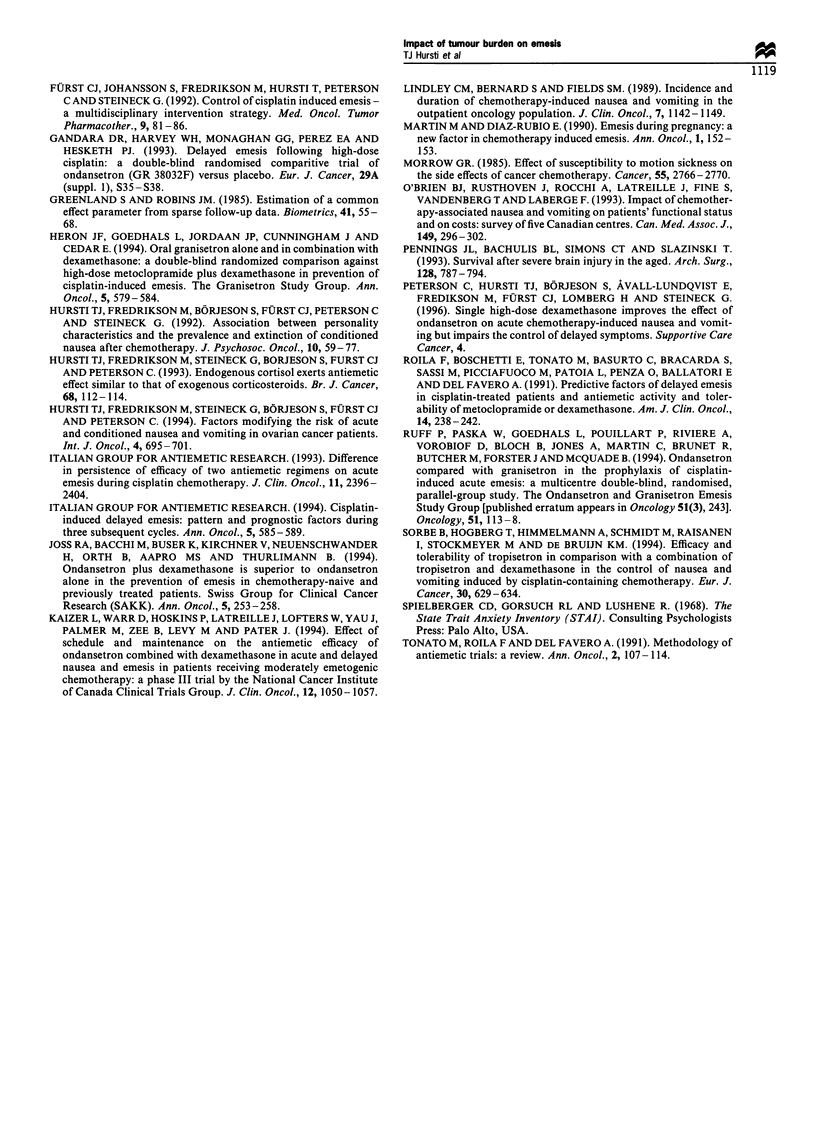# Impact of tumour burden on chemotherapy-induced nausea and vomiting.

**DOI:** 10.1038/bjc.1996.499

**Published:** 1996-10

**Authors:** T. J. Hursti, E. Avall-Lundqvist, S. Börjeson, M. Fredrikson, C. J. Fürst, G. Steineck, C. Peterson

**Affiliations:** Department of Clinical Neuroscience, Karolinska Institute, Stockholm, Sweden.

## Abstract

We investigated how residual tumour burden after cytoreductive surgery was related to the occurrence of acute and delayed nausea and vomiting in 101 ovarian cancer patients receiving their first chemotherapy course. The anti-emetic treatment included ondansetron combined with dexamethasone or placebo. After chemotherapy all patients received ondansetron only for 5 days. Two categories of tumour burden (TB) were formed according to the diameter of the greatest residual tumour (< 2 cm = minimal TB and > or = 2 cm = large TB). Self-reports of nausea and vomiting were obtained for 15 days. Other potential predictor variables were assessed and included in multivariate analyses. Patients with large compared with minimal TB had more delayed emesis, especially on days 2-7. They also had more acute nausea. The aggravating effect associated with large residual TB was more evident in patients > or = 55 years. During the second week after the chemotherapy the occurrence of nausea was higher in patients > or = 55 years than in those < 55 years. This was seen primarily in patients with large residual TB. Predictors for no delayed emesis at all were anti-emetic treatment with dexamethasone, minimal tumour burden, low neuroticism and no history of motion sickness. The increased risk of "persistent' delayed nausea and vomiting seen in older patients with large tumour burden may have important clinical implications and warrants further attention.


British Journal of Cancer (1996) 74, 1114-1119
© 1996 Stockton Press All rights reserved 0007-0920/96 $12.00

### Impact of tumour burden on chemotherapy-induced nausea and vomiting

TJ Hursti[1,2], E Åvall-Lundqvist[3], S Börjeson[4], M Fredrikson[2], CJ Fürst[4], G Steineck[5] and C Peterson[6]

[1]*Department of Clinical Neuroscience, Karolinska Institute, S-171 76 Stockholm, Sweden;* [2]*Department of Clinical Psychology, Uppsala University, Box 1225, S-751 42 Uppsala, Sweden; Departments of* [3]*Gynaecological Oncology,* [4]*Oncology,* [5]*Cancer Epidemiology and* [6]*Clinical Pharmacology, Karolinska Institute and Karolinska Hospital, Radiumhemmet, S-171 76 Stockholm, Sweden.*

**Summary** We investigated how residual tumour burden after cytoreductive surgery was related to the occurrence of acute and delayed nausea and vomiting in 101 ovarian cancer patients receiving their first chemotherapy course. The anti-emetic treatment included ondansetron combined with dexamethasone or placebo. After chemotherapy all patients received ondansetron only for 5 days. Two categories of tumour burden (TB) were formed according to the diameter of the greatest residual tumour (<2 cm = minimal TB and ⩾2 cm = large TB). Self-reports of nausea and vomiting were obtained for 15 days. Other potential predictor variables were assessed and included in multivariate analyses. Patients with large compared with minimal TB had more delayed emesis, especially on days 2–7. They also had more acute nausea. The aggravating effect associated with large residual TB was more evident in patients ⩾55 years. During the second week after the chemotherapy the occurrence of nausea was higher in patients ⩾55 years than in those <55 years. This was seen primarily in patients with large residual TB. Predictors for no delayed emesis at all were anti-emetic treatment with dexamethasone, minimal tumour burden, low neuroticism and no history of motion sickness. The increased risk of 'persistent' delayed nausea and vomiting seen in older patients with large tumour burden may have important clinical implications and warrants further attention.

**Keywords:** delayed emesis; tumour burden; mechanisms

Delayed emesis is a major problem for many patients. It starts by definition 24 h after the beginning of the chemotherapy and may last for several days (Joss *et al.*, 1994; Sorbe *et al.*, 1994). As opposed to the model for acute emesis with an assumed single dominating mechanism (release of 5-HT acting on abdominal 5-$HT_3$ receptors resulting in activation of vagal afferents), several pathways have been proposed for delayed nausea and vomiting. Cerebral oedema, disordered gut function and cell degradation products are factors suggested as related to delayed emesis but the empirical evidence for any mechanism is sparse (Andrews and Davis, 1993).

Identifying predictors for delayed nausea and vomiting may aid in the understanding of the pathogenesis of the disorder and in optimising the anti-emetic treatment. The dose of cisplatin and preceding acute emesis or emesis during previous cycles have been established as prognostic factors for delayed emesis (du Bois *et al.*, 1992; Italian Group for Antiemetic Research, 1994; Roila *et al.*, 1991). High pretreatment noradrenaline excretion as well as low cortisol excretion have been associated with nausea or vomiting occurring more than 24 h after the start of the chemotherapy (Fredrikson *et al.*, 1992, 1994; Hursti *et al.*, 1993). In some studies gender has been reported to affect delayed nausea (Kaizer *et al.*, 1994; Roila *et al.*, 1991) or vomiting (du Bois *et al.*, 1992) but in other reports no significant association was found (Italian Group for Antiemetic Research, 1994; Carmichael *et al.*, 1994; Gandara *et al.*, 1993; Lindley *et al.*, 1989). One study reported an association between previous motion sickness and delayed nausea (Kaizer *et al.*, 1994). Summing up, only a few patient characteristics have so far been found to modify delayed nausea and vomiting. However, most of the studies accomplished were not primarily designed to identify predictors of delayed emesis.

In the light of the suggested mechanisms for delayed emesis, the study of the potential influence of residual tumour burden on chemotherapy-induced nausea and vomiting is warranted. The aim of the present study was to investigate this relation during a 15 day assessment period starting from the day of cisplatin administration of the patients' first chemotherapy cycle.

### Patients and methods

### *Patients*

A total of 101 chemotherapy-naive ovarian cancer patients referred to the Department of Gynaecological Oncology, Radiumhemmet, participated in the study. Exclusion criteria included severe concurrent disease, gastrointestinal obstruction, vomiting and/or having received anti-emetics within 24 h before the start of chemotherapy. All patients had undergone primary cytoreductive surgery about 1 month earlier. Residual tumour burden after completed surgery was estimated by the surgeon. Information about the residual tumour burden was gathered from the surgery records without knowledge of the patients' scoring of nausea and vomiting. Tumour burden was first classified into four categories according to the diameter of the greatest residual tumour: (1) from no visible tumour to less than 2 cm ($n=60$); (2) 2–5 cm ($n=11$); (3) 5–10 cm ($n=6$); and (4) larger than 10 cm ($n=24$). In the statistical analyses the first group (<2 cm), termed 'minimal tumour burden' was compared with the rest of the patients (⩾2 cm), termed 'large tumour burden'. The median age was 54 years (range 18–76 years).

The chemotherapy included cisplatin (50 mg $m^{-2}$) combined with either doxorubicin (50 mg $m^{-2}$) during a single day ($n=33$) or doxorubicin (40 mg $m^{-2}$) and melphalan (0.4 mg $kg^{-1}$) on the day before cisplatin ($n=68$). As anti-emetic medication, patients received ondansetron 8 mg i.v. × 3, on both chemotherapy days (applies for the 2 day treatment) and were randomised to combine ondansetron either with dexamethasone (20 mg i.v. × 1) or placebo given 6 h after the cisplatin infusion was started. Additionally, all patients received ondansetron (8 mg orally × 3) daily for 5 days after the chemotherapy. Results concerning the anti-emetic trial (dexamethasone *vs* placebo) will be reported

Correspondence: TJ Hursti, Department of Clinical Psychology, Uppsala University, Box 1225, S-751 42 Uppsala, Sweden
Received 31 October 1995; revised 9 April 1996; accepted 29 April 1996

elsewhere (Peterson *et al.*, 1996). The study was approved by the ethics committee at the Karolinska Hospital and consent was obtained from all patients.

### *Methods*

Nausea and vomiting were self-recorded by the patients daily starting on the day of cisplatin administration (=day 0) and continuing on days 1 to 14. Nausea was registered on a four-grade scale (none, mild, moderate or severe) and vomiting as the number of emetic episodes. An emetic episode was defined as a single vomit or retch or any number of continuous vomits and/or retches. The patients were instructed to fill in the registration form every morning as an average estimation of the symptoms during the previous 24 h period. In addition, on arrival at the hospital on the day before their first chemotherapy course, an assessment of the patients' functional status and general well-being during the preceding week was made. The patients were requested to report, using a 100 mm visual analogue scale (VAS), if they had been bothered by e.g. nausea, vomiting, sleeping disorders, pain and anxiety. Additionally 12 aspects of daily life were investigated with this method.

Some other characteristics previously reported as associated with chemotherapy-related nausea and vomiting were also assessed (Andrykowski and Gregg, 1992; Hursti *et al.*, 1992, 1994; Martin and Diaz-Rubio, 1990; Morrow, 1985). These were age, history of nausea and vomiting in general and in specific situations (motion sickness, nausea during pregnancy, nausea related to alcohol consumption), trait and state anxiety (Spielberger *et al.*, 1968), neuroticism (Eysenck and Eysenck, 1964) and autonomic perception (Borcovec, 1976). The purpose was to identify possible confounding factors for the findings concerning tumour burden.

### *Statistics*

In the statistical analyses patients with minimal tumour burden (i.e. <2 cm) were compared with those having large tumour burden (i.e. ⩾2 cm). Ratio of proportions (RP) was used to describe the association between the studied factors and nausea or vomiting. It was calculated as the ratio between the proportions of patients with no nausea or no emetic episodes (i.e. complete response) in the groups of interest. To adjust for the differences in anti-emetic treatment and the possible confounding effect from another studied factor, data were stratified and a weighted ratio of proportions was computed with a method described by Ahlbom (1990). Calculation of 95% confidence intervals was performed based on a variance described by Greenland and Robins (1985). Ratio of proportions provides a very comprehensible measure of effect. However, since the method we used allowed a simultaneous adjustment only for a limited number of other variables, we also performed logistic regression analyses with tumour burden, all the patient characteristics (see Methods) and anti-emetic treatment entered in one block. As a complement to the day-by-day analyses, we sought to predict the total anti-emetic response in the entire delayed phase. For that purpose the outcome was defined as a binary response based on whether the patient had experienced emetic episodes and nausea, respectively, during any of the days 1–14). Associations between tumour burden and other patient characteristics were analysed by $\chi^2$ test where continuous variables were dichotomised by a median-split approach. Prechemotherapy VAS ratings were analysed by Student's *t*-test and $\chi^2$ test.

### Results

With the exception of age, groups with minimal compared with large tumour burden were well balanced concerning patient and treatment characteristics (all $\chi^2(1)<1.4$; NS) (Table I). Sixty-eight per cent of the patients with large tumour burden were above the median age (i.e. ⩾55 years) as compared with 38% among patients with minimal tumour burden ($\chi^2(1)=8.7$; $P=0.003$). Nausea and vomiting were similar in the groups of patients having received their chemotherapy on a single day compared with a 2 day treatment.

### *Effects on emetic episodes*

Figure 1 displays the proportions of patients free from emetic episodes as a function of tumour burden. A quite consistent trend showing that the prevalence of emetic episodes was higher among patients with large tumour burden (i.e. ⩾2 cm in greatest diameter) was observed throughout the assessment period. Ratios of proportions, adjusted for age and anti-emetic treatment, were significant for days 3 [RP (with 95% confidence interval) 1.4 (1.1–1.9)], 4 [RP 1.2 (1.0–1.4)] and 5 [RP 1.2 (1.0–1.5)]. Restricting the analysis to older (⩾55 years) patients revealed an even more marked association (Figure 2). Ratios of proportions adjusted for anti-emetic

**Table 1** Patient and treatment characteristics in groups of patients categorised by the diameter of the greatest residual tumour (<2 cm *vs* ⩾2 cm)

| *Variable* | *Patients with tumour<2 cm* | *Patients with tumour⩾2 cm* | P |
|---|---|---|---|
| Number of patients | 60 | 41 | |
| Antiemetics | | | NS |
| Ondansetron and dexamethasone | 34 | 19 | |
| Ondansetron and placebo | 26 | 22 | |
| Chemotherapy given on | | | NS |
| A single day | 17 | 16 | |
| Two days | 43 | 25 | |
| Age (mean) | 51.5 | 59.1 | <0.001 |
| Previous history of (%) | | | |
| Nausea in general | 70 | 71 | N.S. |
| Vomiting in general | 58 | 54 | N.S. |
| Motion sickness | 40 | 49 | N.S. |
| Nausea during pregnancy | 59 | 47 | N.S. |
| Nausea related to alcohol consumption | 12 | 7 | N.S. |
| Personality (mean scores on the inventories) | | | |
| State anxiety | 45.3 | 44.6 | N.S. |
| Trait anxiety | 35.5 | 35.7 | N.S. |
| Neuroticism | 6.1 | 5.2 | N.S. |
| Autonomic perception | 67.6 | 63.2 | N.S. |

**Figure 1** Proportions of patients free from emetic episodes as a function of residual tumour burden (see Methods). □, Minimal tumour; ■, large tumour.

**Figure 2** Proportions of patients free from emetic episodes as a function of residual tumour burden among those aged 55 years or more. □, Minimal tumour and ⩾55 years; ■, large tumour and ⩾55 years.

**Figure 3** Proportions of patients free from nausea as a function of age. □, <55 years; ■, ⩾55 years.

**Figure 4** Proportions of patients free from nausea as a function of age among those having large residual tumour burden. □, <55 years and large tumour; ■, ⩾55 years and large tumour.

**Figure 5** Proportions of patients free from emetic episodes as a function of residual tumour burden and/or age. □, <55 years and minimal tumour; ◆, ⩾55 years or large tumour; ■, ⩾55 years and large tumour.

**Figure 6** Proportions of patients free from nausea as a function of residual tumour burden and/or age. □, <55 years and minimal tumour; ◆, ⩾55 years or large tumour; ■, ⩾55 years and large tumour.

treatment reached significance for days 3 to 7 ranging from 1.2 to 1.5. Among younger patients, the size of the remaining tumour was not significantly associated with emetic episodes.

A statistically non-significant initial trend for more frequent emetic episodes in older patients was observed. Ratio of proportions adjusted for tumour burden and anti-emetic treatment was 1.4 (0.9–2.2) for the cisplatin day and 1.2 (0.9–1.8) for day 1 after chemotherapy.

The logistic regression analyses with all the predictor variables entered in one block showed that tumour burden significantly predicted emetic episodes during days 2–3 and 5–7 ($P<0.05$) and that the prediction was marginally significant for day 4 ($P=0.07$) and day 8 ($P=0.055$). The same method was used to predict the total anti-emetic response in the delayed phase (i.e. no delayed emesis on any day) and resulted in the following statistically significant predictors: anti-emetic treatment with dexamethasone ($P=0.016$), minimal tumour burden ($P=0.021$), low neuroticism ($P=0.03$) and no previous history of motion sickness ($P=0.033$). Among patients with large tumour burden 70.7% experienced delayed emesis compared with 46.7% of those with minimal tumour burden [RP 1.5 (1.1–2.1)]. The analysis of total anti-emetic response relies actually on days 1 to 4 since no patient had her first day of delayed emesis later than day 4.

*Effects on nausea*

Significantly more patients with large tumour burden reported nausea on the chemotherapy day compared with those with minimal tumour burden. The ratio of proportions adjusted for age and anti-emetic treatment was 2.0 (1.0–4.1). In the delayed phase (days 1–14) no significant association was seen.

The overall effect of age on nausea during the monitoring period is presented in Figure 3. From about 1 week after the chemotherapy and onwards an increasing trend for more frequent delayed nausea in older (⩾55 years) patients was observed. For days 9–13, ratios of proportions adjusted for tumour burden and anti-emetic treatment were 1.2–1.3 with the 95% confidence interval separated from 1.0. This pattern was evident primarily in patients with large tumour burden (Figure 4). In this group, ratios of proportions adjusted for anti-emetic treatment reached significance for day 7 [RP 1.7 (1.1–2.6)], day 9 [RP 1.5 (1.2–2.0)], day 10 [RP 1.5 (1.1–1.9)], day 11 [RP 1.5 (1.2–2.0)], day 12 [RP 1.3 (1.1–1.6)] and day 13 [RP 1.4 (1.1–1.8)]. In patients with minimal tumour burden, no significant association between age and nausea was observed during the study days.

The logistic regression analyses confirmed that tumour burden predicted nausea on the chemotherapy day ($P=0.013$) but not in the delayed phase. Age predicted nausea on days 9–12 ($P<0.05$). In the analysis using the total anti-emetic response as the outcome (i.e. no delayed nausea on any day), history of motion sickness was the only significant predictor variable ($P=0.018$). Only 18 of the 101 participating patients totally escaped from delayed nausea and 17 of these lacked the previous history of motion sickness. The analysis of total anti-emetic response describes what happens during days 1–5 since no patient had her first day of delayed nausea later than day 5.

*Interaction between tumour burden and age*

To illustrate interaction between tumour burden and age further we compared patients with both the risk factors (i.e. tumour burden ⩾2 cm and age ⩾55 years) with those with none. Patients with only one of the two characteristics were treated as one group. The results are presented in Figure 5 (emetic episodes) and Figure 6 (nausea). In both cases the group with large residual tumour burden and higher age clearly differs from the other two groups. The group with only one of the risk factors shows a greater resemblance to those with no risk factor than those with two.

*Symptoms in the preceding week*

The overall rating levels concerning the functional status during the week preceding the chemotherapy were similar to those obtained in a previous study with ovarian cancer patients (Fürst *et al.*, 1992). However, the results indicated a more compromised well-being for the patients with large residual tumour burden compared with those with minimal tumour burden (Table II). Significant differences were found for vomiting, appetite, fatigue, general well-being and (nearly significantly) strength. The patients with large tumour burden were also less satisfied with the information they received concerning their illness and its treatment. We also analysed nausea and vomiting concerning the pure prevalence by dichotomising the ratings in 'not bothered at all' (VAS=0) and 'bothered' (VAS 1–100). Vomiting but not nausea was more common in patients with large compared with minimal tumour burden (vomiting, $\chi^2=7.3$, $P<0.01$; nausea, $\chi^2=0.1$, NS). Age was not related to nausea and vomiting during the prechemotherapy period but the older patients complained more about lack of strength and difficulties in relaxation when compared with the younger ones (Table II).

**Table 2** Functional status during the week preceding the chemotherapy start

| Variable | *Tumour size* <2 cm | *Tumour size* ⩾2 cm | P | *Age* <55 years | *Age* ⩾55 years | P |
|---|---|---|---|---|---|---|
| Nausea | 8.8 | 10.6 | >0.10 | 11.4 | 7.7 | >0.10 |
| Vomiting | 2.0 | 6.7 | 0.014 | 2.8 | 4.9 | >0.10 |
| Pain | 19.3 | 26.1 | >0.10 | 20.2 | 23.9 | >0.10 |
| Lack of appetite | 19.6 | 32.5 | 0.014 | 23.6 | 23.0 | >0.10 |
| Fatigue | 33.4 | 45.9 | 0.017 | 33.8 | 43.0 | 0.076 |
| Lack of strength | 33.5 | 43.8 | 0.053 | 31.4 | 43.9 | 0.015 |
| Difficulties in physical activity | 23.4 | 31.5 | 0.084 | 24.2 | 29.1 | >0.10 |
| Feeling down | 26.8 | 36.8 | 0.069 | 30.0 | 31.8 | >0.10 |
| Anger | 23.1 | 30.2 | >0.10 | 28.6 | 23.3 | >0.10 |
| Anxiety | 37.3 | 42.1 | >0.10 | 38.6 | 39.9 | >0.10 |
| Difficulties in relaxation | 35.1 | 44.6 | 0.080 | 33.2 | 44.6 | 0.032 |
| Difficulties in concentration | 28.6 | 36.8 | 0.086 | 30.7 | 33.1 | >0.10 |
| Sleeping disorders | 47.5 | 51.5 | >0.10 | 49.2 | 48.7 | >0.10 |
| Disturbed family interaction | 8.1 | 7.5 | >0.10 | 9.0 | 6.7 | >0.10 |
| Disturbed other social interaction | 15.7 | 17.1 | >0.10 | 15.6 | 16.9 | >0.10 |
| Unsatisfied with information | 8.5 | 16.0 | 0.038 | 12.3 | 10.7 | >0.10 |
| Lack of general well-being | 41.9 | 51.9 | 0.037 | 43.8 | 48.0 | >0.10 |

Mean ratings on a 100 mm visual analogue scale for patients categorised by the diameter of the greatest residual tumour (<2 cm *vs* ⩾2 cm) and by age (<55 years *vs*⩾55 years). Low ratings correspond to unaffected well-being.

### Discussion

Our results indicate an association between residual tumour burden and chemotherapy-induced nausea and vomiting. Thus, patients with tumour burden $\geqslant 2$ cm in greatest diameter compared with those with minimal tumour burden reported more frequent emetic episodes in the delayed phase and also, somewhat less articulated, in the acute phase. They experienced more often acute but not delayed nausea. Compared with younger ones, older patients reported more frequent nausea during the second week after the chemotherapy. They also tended to have more frequent emetic episodes during the first days of the chemotherapy cycle. The anti-emetic response for delayed emesis (no delayed emesis) was predicted by anti-emetic treatment with dexamethasone, minimal tumour burden, low neuroticism and the lack of history of motion sickness. Likewise, the anti-emetic response for delayed nausea was predicted by the lack of history of motion sickness.

There may be several co-existing factors explaining our results. The majority of patients categorised as having large residual tumour burden had an intra-abdominal tumour $>10$ cm in greatest diameter. Hypothetically such a tumour mass may exert a mechanical pressure on the gut leading to nausea and vomiting. Alternatively, spontaneous or chemotherapy-induced tumour necrosis may cause a release of substances (e.g. prostaglandins or cytokines) from the tumour and influence nausea and vomiting (Andrews and Davis, 1993).

During the week preceding the chemotherapy the patients with large residual tumour burden reported vomiting significantly more often accompanied by loss of appetite and strength, increased fatigue and generally inferior well-being compared with patients with minimal tumour burden. Hypothetically, this could be explained by the above-mentioned mechanisms. As demonstrated in Figures 1 and 2 it seems that in the group of patients with large residual tumour, the rate of complete response does not improve after day 9. This observed difference may to some extent reflect prechemotherapy differences. However, it should be pointed out that none of the patients experienced vomiting 24 h before the start of the chemotherapy.

Older patients, particularly those with large residual tumour burden, reported more frequent side-effects in the delayed phase compared with younger patients. This is in contrast to some previous studies showing an inverse relationship between age and acute nausea and vomiting (Tonato *et al.*, 1991). The association between age and emesis has not been thoroughly studied during the delayed phase. To our knowledge no previous report has shown age differences in delayed nausea or vomiting (Carmichael *et al.*, 1994; du Bois *et al.*, 1992; Gandara *et al.*, 1993; Italian Group for Antiemetic Research, 1994; Kaizer *et al.*, 1994; Lindley *et al.*, 1989; Roila *et al.*, 1991). Interestingly, in more recent studies, the evidence provided for an inverse relationship between age and emesis in the acute phase has not been consistent (de Wet *et al.*, 1993; Heron *et al.*, 1994; Italian Group for Antiemetic Research, 1993; Ruff *et al.*, 1994). This may be related to the introduction of new anti-emetic regimens. The mechanisms mediating the effect of age on nausea and vomiting are not known. However, the association is most likely multifactorially determined and hence modified by several variables.

The combined aggravating effects of tumour burden and age in the delayed phase may also be conceived of as a more general age-dependent problem of recovery (Erschler and Balducci, 1994). In general, elderly patients recover more slowly after, for example, surgery or an injury (Artinian *et al.*, 1993; Pennings *et al.*, 1993). The effect of cancer treatment is also less favourable for older patients compared with younger patients (Alberts *et al.*, 1993). As demonstrated in Figure 4 older patients with tumour seem to recover more slowly from the chemotherapy compared with younger patients (slope of the curve is less steep for older patients).

In conclusion, monitoring nausea and vomiting up to 2 weeks after chemotherapy revealed that while the delayed symptoms decreased exponentially during the first week, there was practically no further improvement during the second week. The results suggest that older patients with large residual tumour burden are at increased risk of this 'persistent' delayed nausea and vomiting. Also, earlier in the delayed phase these patients suffered from more frequent emetic episodes. Large tumour burden was associated with compromised well-being already before the treatment started. This is decremental since persistent nausea and vomiting may lead to a descending spiral with further worsening of functional status (O'Brien *et al.*, 1993). The impact of 'persistent' delayed nausea on delivered dose intensity of chemotherapy given with a curable intent and well-being when given in a palliative setting warrants further attention.

**Acknowledgements**
This study was supported by grants from the Swedish Cancer Society, King Gustaf V Jubilee Fund and Glaxo Sweden AB.

### References

AHLBOM A. (1990). *Biostatistik för epidemiologer* (*Biostatistics for Epidemiologists*). Studentlitteratur: Lund.

ALBERTS DS, DAHLBERG S, GREEN SJ, GARCIA D, HANNIGAN EV, O'TOOLE R, STOCK NOVACK D, SURWIT EA, MALVIYA VK AND JOLLES CJ. (1993). Analysis of patient age as an independent prognostic factor for survival in a phase III study of cisplatin–cyclophosphamide versus carboplatin–cyclophosphamide in stages III (suboptimal) and IV ovarian cancer. A Southwest Oncology Group study. *Cancer*, **71**, 618–627.

ANDREWS PLR AND DAVIS CJ. (1993). The mechanism of emesis induced by anti-cancer therapies. In *Emesis in Anti-cancer Therapy, Mechanisms and Treatment*, Andrews PLR and Sanger CJ. (eds) pp. 113–161. Chapman & Hall: London.

ANDRYKOWSKI MA AND GREGG ME. (1992). The role of psychological variables in post-chemotherapy nausea: anxiety and expectation. *Psychosom. Med.*, **54**, 48–58.

ARTINIAN NT, DUGGAN C AND MILLER P. (1993). Age differences in patient recovery patterns following coronary artery bypass surgery. *Am. J. Crit. Care*, **2**, 453–461.

BORCOVEC TD. (1976). Physiological and cognitive processes in the regulation of anxiety. In *Consciousness and Self-regulation. Advances in Research*, Schwartz GE and Shapiro S. (eds) pp. 261–312. Plenum Press: New York.

CARMICHAEL J, BESSEL EM, HARRIS AL, HUTCHEON AW, DAWES PJ AND DANIELS S. (1994). Comparison of granisetron alone and granisetron plus dexamethasone in the prophylaxis of cytotoxic-induced emesis. *Br. J. Cancer*, **70**, 1161–1164.

DE WET M, FALKSON G AND RAPOPORT BL. (1993). Repeated use of granisetron in patients receiving cytostatic agents. *Cancer*, **71**, 4043–4049.

DU BOIS A, MEERPOHL HG, VACH W, KOMMOSS FG, FENZL E AND PFLEIDERER A. (1992). Course, patterns, and risk-factors for chemotherapy-induced emesis in cisplatin-pretreated patients: a study with ondansetron. *Eur. J. Cancer*, **28**, 450–457.

ERSCHLER WB AND BALDUCCI L. (1994). Treatment considerations for older patients with cancer. *In Vivo*, **8**, 737–744.

EYSENCK HJ AND EYSENCK SBG. (1964). *Manual of the Eysenck Personality Inventory*. University of London Press: London.

FREDRIKSON M, HURSTI T, FURST CJ, STEINECK G, BORJESON S, WIKBLOM M AND PETERSON C. (1992). Nausea in cancer chemotherapy is inversely related to urinary cortisol excretion. *Br. J. Cancer*, **65**, 779–780.

FREDRIKSON M, HURSTI T, STEINECK G, FÜRST CJ, BÖRJESON S AND PETERSON C. (1994). Delayed emesis of chemotherapy is augmented by high levels of endogenous noradrenaline. *Br. J. Cancer*, **70**, 642–645.

FÜRST CJ, JOHANSSON S, FREDRIKSON M, HURSTI T, PETERSON C AND STEINECK G. (1992). Control of cisplatin induced emesis – a multidisciplinary intervention strategy. *Med. Oncol. Tumor Pharmacother.*, **9**, 81–86.

GANDARA DR, HARVEY WH, MONAGHAN GG, PEREZ EA AND HESKETH PJ. (1993). Delayed emesis following high-dose cisplatin: a double-blind randomised comparitive trial of ondansetron (GR 38032F) versus placebo. *Eur. J. Cancer*, **29A** (suppl. 1), S35–S38.

GREENLAND S AND ROBINS JM. (1985). Estimation of a common effect parameter from sparse follow-up data. *Biometrics*, **41**, 55–68.

HERON JF, GOEDHALS L, JORDAAN JP, CUNNINGHAM J AND CEDAR E. (1994). Oral granisetron alone and in combination with dexamethasone: a double-blind randomized comparison against high-dose metoclopramide plus dexamethasone in prevention of cisplatin-induced emesis. The Granisetron Study Group. *Ann. Oncol.*, **5**, 579–584.

HURSTI TJ, FREDRIKSON M, BÖRJESON S, FÜRST CJ, PETERSON C AND STEINECK G. (1992). Association between personality characteristics and the prevalence and extinction of conditioned nausea after chemotherapy. *J. Psychosoc. Oncol.*, **10**, 59–77.

HURSTI TJ, FREDRIKSON M, STEINECK G, BORJESON S, FURST CJ AND PETERSON C. (1993). Endogenous cortisol exerts antiemetic effect similar to that of exogenous corticosteroids. *Br. J. Cancer*, **68**, 112–114.

HURSTI TJ, FREDRIKSON M, STEINECK G, BÖRJESON S, FÜRST CJ AND PETERSON C. (1994). Factors modifying the risk of acute and conditioned nausea and vomiting in ovarian cancer patients. *Int. J. Oncol.*, **4**, 695–701.

ITALIAN GROUP FOR ANTIEMETIC RESEARCH. (1993). Difference in persistence of efficacy of two antiemetic regimens on acute emesis during cisplatin chemotherapy. *J. Clin. Oncol.*, **11**, 2396–2404.

ITALIAN GROUP FOR ANTIEMETIC RESEARCH. (1994). Cisplatin-induced delayed emesis: pattern and prognostic factors during three subsequent cycles. *Ann. Oncol.*, **5**, 585–589.

JOSS RA, BACCHI M, BUSER K, KIRCHNER V, NEUENSCHWANDER H, ORTH B, AAPRO MS AND THURLIMANN B. (1994). Ondansetron plus dexamethasone is superior to ondansetron alone in the prevention of emesis in chemotherapy-naive and previously treated patients. Swiss Group for Clinical Cancer Research (SAKK). *Ann. Oncol.*, **5**, 253–258.

KAIZER L, WARR D, HOSKINS P, LATREILLE J, LOFTERS W, YAU J, PALMER M, ZEE B, LEVY M AND PATER J. (1994). Effect of schedule and maintenance on the antiemetic efficacy of ondansetron combined with dexamethasone in acute and delayed nausea and emesis in patients receiving moderately emetogenic chemotherapy: a phase III trial by the National Cancer Institute of Canada Clinical Trials Group. *J. Clin. Oncol.*, **12**, 1050–1057.

LINDLEY CM, BERNARD S AND FIELDS SM. (1989). Incidence and duration of chemotherapy-induced nausea and vomiting in the outpatient oncology population. *J. Clin. Oncol.*, **7**, 1142–1149.

MARTIN M AND DIAZ-RUBIO E. (1990). Emesis during pregnancy: a new factor in chemotherapy induced emesis. *Ann. Oncol.*, **1**, 152–153.

MORROW GR. (1985). Effect of susceptibility to motion sickness on the side effects of cancer chemotherapy. *Cancer*, **55**, 2766–2770.

O'BRIEN BJ, RUSTHOVEN J, ROCCHI A, LATREILLE J, FINE S, VANDENBERG T AND LABERGE F. (1993). Impact of chemotherapy-associated nausea and vomiting on patients' functional status and on costs: survey of five Canadian centres. *Can. Med. Assoc. J.*, **149**, 296–302.

PENNINGS JL, BACHULIS BL, SIMONS CT AND SLAZINSKI T. (1993). Survival after severe brain injury in the aged. *Arch. Surg.*, **128**, 787–794.

PETERSON C, HURSTI TJ, BÖRJESON S, ÅVALL-LUNDQVIST E, FREDIKSON M, FÜRST CJ, LOMBERG H AND STEINECK G. (1996). Single high-dose dexamethasone improves the effect of ondansetron on acute chemotherapy-induced nausea and vomiting but impairs the control of delayed symptoms. *Supportive Care Cancer*, **4**.

ROILA F, BOSCHETTI E, TONATO M, BASURTO C, BRACARDA S, SASSI M, PICCIAFUOCO M, PATOIA L, PENZA O, BALLATORI E AND DEL FAVERO A. (1991). Predictive factors of delayed emesis in cisplatin-treated patients and antiemetic activity and tolerability of metoclopramide or dexamethasone. *Am. J. Clin. Oncol.*, **14**, 238–242.

RUFF P, PASKA W, GOEDHALS L, POUILLART P, RIVIERE A, VOROBIOF D, BLOCH B, JONES A, MARTIN C, BRUNET R, BUTCHER M, FORSTER J AND MCQUADE B. (1994). Ondansetron compared with granisetron in the prophylaxis of cisplatin-induced acute emesis: a multicentre double-blind, randomised, parallel-group study. The Ondansetron and Granisetron Emesis Study Group [published erratum appears in *Oncology* **51(3)**, 243]. *Oncology*, **51**, 113–8.

SORBE B, HOGBERG T, HIMMELMANN A, SCHMIDT M, RAISANEN I, STOCKMEYER M AND DE BRUIJN KM. (1994). Efficacy and tolerability of tropisetron in comparison with a combination of tropisetron and dexamethasone in the control of nausea and vomiting induced by cisplatin-containing chemotherapy. *Eur. J. Cancer*, **30**, 629–634.

SPIELBERGER CD, GORSUCH RL AND LUSHENE R. (1968). *The State Trait Anxiety Inventory (STAI)*. Consulting Psychologists Press: Palo Alto, USA.

TONATO M, ROILA F AND DEL FAVERO A. (1991). Methodology of antiemetic trials: a review. *Ann. Oncol.*, **2**, 107–114.